# Investigation of Combined Aging and Mullins Stress Softening of Rubber Nanocomposites

**DOI:** 10.3390/polym16223141

**Published:** 2024-11-11

**Authors:** Mohamed Bakar, Marta Sola-Wdowska, Małgorzata Przybyłek, Anita Białkowska, Iwona Zarzyka, Barbora Hanulikova, Milan Masař

**Affiliations:** 1 Faculty of Applied Chemistry, Radom University, 26-600 Radom, Poland; m.przybylek@uthrad.pl (M.P.); a.bialkowska@uthrad.pl (A.B.); 2 Faculty of Chemistry, Rzeszów University of Technology, 35-959 Rzeszów, Poland; izarzyka@prz.edu.pl; 3 Center of Polymer Systems, Tomas Bata University in Zlin, 760 01 Zlín, Czech Republic; hanulikova@utb.cz (B.H.); masar@utb.cz (M.M.)

**Keywords:** rubber nanocomposites, Mullins effect, aging, mechanical properties, thermal properties, structure, morphology

## Abstract

The present study investigated the effects of thermal aging, ultraviolet radiation (UV), and stress softening on the performance properties of rubber modified with Cloisite Na^+^ or Cloisite 20A. Tensile strength (TS), strain at break (SB), modulus, and the retention coefficient were measured before and after aging. Results showed that TS and SB decreased by about 50% after 7 days of aging for all tested samples due to the breakage of the chemical bonds between rubber and nanoparticles. The modulus at 300% elongation increased by 20%, 15%, and 7% after thermal aging for the unmodified sample, nanocomposites with Cloisite Na^+^, and Cloisite 20A, respectively. The shape retention coefficient of all samples was not affected by heat, except for the virgin rubber sample, which exhibited a decrease of about 15% under thermal aging. The virgin matrix and nanocomposites showed different values of aging coefficient during thermal aging and UV radiation. The dissipated energy of samples that were aged after stretching was slightly higher than that of samples that were aged after stretching due to the breakdown of the bonds within the nanocomposites. Loading-reloading energy results showed that the level of stress softening was lower when Mullins was applied after the aging of the samples. Differential scanning calorimetry results indicated a slight decrease in T_g1_ in the aged and stretched samples and an increase in the temperature of the first endothermic peak due to the addition of nanofillers in the stretched and aged samples. Thermogravimetric analysis revealed that all tested samples exhibited similar thermograms, regardless of their state of stretching or aging. Scanning electron microscopy analysis showed that the fracture surface of the virgin unaged sample was rough with some holes, while it was flatter and less rough after aging.

## 1. Introduction

The field of rubber nanocomposites has received considerable attention from both academics and industrialists in recent decades due to their improved mechanical performance and thermal stability. The development and applications of rubber nanocomposites have been the subject of many reviews and research works [1,2,3,4,5,6,7,8]. The properties of rubber nanocomposites have been shown to depend on the type and content of nanofillers, as well as the dispersion methods. Good dispersion leads to greater interfacial interactions with the rubber matrix compared to microfillers, which results in the expected reinforcing effect. Additionally, the amplitude and time of mixing of nanoparticles with the matrix have been shown to affect the mechanical properties of polymer nanocomposites [9,10,11].

Due to the specific application conditions of filled and unfilled rubbers, various studies have been concerned with their aging behavior [12,13,14,15,16,17]. Choudhury et al. [12] studied the effect of accelerated heat aging on the properties of hydrogenated nitrile rubber. Results showed that heat aging led to crosslinking reactions and embrittlement of the samples. However, the incorporation of nanoclays restricted the degradation of rubber nanocomposites with improved ductility. Bellas et al. [13] studied the aging of styrene butadiene rubber (SBR) reinforced with organoclays at 70 °C at different times. The elongation at break of the aged nanocomposite samples without antioxidants decreased, whereas the modulus and hardness increased with aging time. These results were attributed to main-chain scission and crosslink formation. Chakraborty et al. [14] studied the effect of thermal aging at 130 °C for 30 h and thermo-oxidative aging at 105 °C for 7 days on the properties of SBR nanocomposite. The results showed that the retention of the properties of the nanocomposites under both aging conditions was better than that of rubber filled with carbon black. Furthermore, it was confirmed that the thermal degradation rate of hydrogenated nitrile butadiene rubber/nanoclay/CNTs nanocomposites was lower than that of HNBR/nanoclay [15]. This was due to the reduction in the diffusion rate of the degradation products induced by the clay and carbon nanotubes (CNT) nanofiller systems.

Various interpretations have been put forward to explain the aging processes of polymer nanocomposites as well as ways to reduce the degradation of the latter. Chain scission and crosslink formation are the most accepted mechanisms that lead to the deterioration of mechanical properties [12,13,18,19]. It has also been demonstrated that clay nanoplatelets act as a barrier against the diffusion of degradation products, thus delaying the degradation of nanocomposites [15,19,20]. It is well documented that a very good dispersion of nanofillers combined with interfacial adhesion with the rubber matrix significantly contributes to the reduction in the degradation of rubber nanocomposites.

Although rubber products are employed in various fields with combined external parameters, relatively few studies have been devoted to aging under stress or strain [21,22,23,24,25,26]. Various interpretations have been proposed to explain the simultaneous effects of aging and applied stress on the performance of rubber materials. It was demonstrated that aging and mechanical tension resulted in a decrease in the crosslink density in silicon rubber samples, unlike thermal aging, which showed the opposite effect [21]. On the contrary, a constantly applied compressive stress causes the breakage of bonds between the natural rubber chains and a decrease in crosslink density [22]. Similar results were obtained by Zheng et al. [23], where crosslinking, chain scission, and oxidation reactions were predominant in the compressive stress relaxation of butadiene rubber samples during thermo-oxidative aging. Li et al. [24] also confirmed that compressive stress and thermo-oxidative aging led to the oxidation of ethylene–propylene–diene monomer (EPDM) rubber structure with the formation of carbonyl groups. In a separate study, it was found that both elevated temperature and applied strain accelerated the aging process of hydrogenated nitrile butadiene rubber (HNBR) [25], while Lou et al. [26] discovered that compressive strain slowed the thermo-oxidative aging reaction of silicone rubber foam. A previous study on the aging of carbon black-filled rubber showed that the oxidative crosslinking of rubber subjected to deformation was significantly accelerated with acceleration increasing with increasing deformation and decreasing temperatures [27].

However, rubber products previously subjected to aging and then subjected to constant stress or strain have been neglected. Quang et al. [28] studied the effect of thermal aging and cyclic tensile loading on the mechanical properties of natural rubber (NR). The tensile strength of NR samples aged at 70 °C remained unchanged, most probably due to strain-induced crystallization, while at 110 °C, both the tensile strength and fatigue resistance of the material reduced drastically. Another study investigated carbon black-filled polychloroprene rubber samples subjected to tensile cycling loading and thermo-oxidative aging. The results showed that aging significantly affected the stress relaxation of the samples due to changes in the network [29].

Numerous researchers have attempted to model the Mullins effect over the past few decades. This phenomenon, known as stress softening, is often accompanied by inelastic effects, such as induced anisotropy, residual strain, and permanent set in loaded rubber samples. Different constitutive models based mostly on pseudo-elastic and damage theories have been used to analyze Mullins stress softening with permanent set and induced anisotropy in filled rubbers [30,31,32,33]. Good agreement was obtained with the experimental data. The complex phenomena and recovery were also investigated. Chu et al. [34] studied the anisotropy and the recovery of filled rubbers. They confirmed that the recovery depends on the annealing time and temperature. However, Merckel et al. [35] captured the anisotropy induced in a wide range of rubber-filled materials. The data were obtained from both uniaxial and biaxial loading tests. However, Fazekas and Goda [36] improved the hyper-visco-pseudo-elastic constitutive equation to model the Mullins effect of carbon black-filled ethylene–propylene–diene monomer rubber by taking into account the effects of temperature and the residual strain effect.

Rubber materials are often used in applications where aging is combined with applied stress or strain, causing permanent deformation of the part and, hence, premature failure. Most of the studies concerned the simultaneous effects of aging and stress or strain on the material properties. The objective of the present work was to study the separate effects and order of effects of aging and cyclic stretching on the performance properties of rubber nanocomposites.

## 2. Materials and Method

### 2.1. Materials

The following ingredients were used to prepare the virgin rubber matrix and rubber nanocomposites containing two different nanoclays:

#### 2.1.1. Base Materials

- KER N-29 (acrylonitrile-butadiene rubber) from Synthos Rubbers, Oświęcim, Poland.

It contains 29% acrylonitrile, has a tensile strength of 20 MPa, and an elongation at break equivalent to 420% according to ASTM D412.

- SVR-3L Natural Rubber–obtained from Dau Tieng Rubber Corporation, Vietnam. It shows high elasticity and elongation at break and good aging resistance.

#### 2.1.2. Nanofillers

- Cloisite 20A–layered aluminosilicate modified with quaternary ammonium salts;
- Cloisite Na^+^: natural montmorillonite.

#### 2.1.3. Other Ingredients in the Formulation

- Zinc oxide white powder used as an accelerator from Huta Będzin, Będzin, Poland;
- Glyceryl tristearate: a mixture of palmitic and stearic acids and a small amount of unsaturated acids, purchased from POCH SA, Gliwice;
- Santicizer 261A–alkyl (C7–C9) benzyl phthalate from Brenntag, Kędzierzyn Koźle, Poland;
- Brown factice–used as a softener of mixtures from Kodrewex Company, Gomunice, Poland;
- Chalk–calcium salt with carbonic acid, mainly used as a filler from Polclac, Łódź, Poland;
- Wax (Protector G 35 WP)–mainly used to reduce friction and improve lubricity (Paramelt BV Costerstraat, Heerhugowaard, The Netherlands);
- Tetramethylthiuram disulfide (Accelerator T) and benzotriazole disulfide (DM Accelerator) were purchased from Rubber Industry in Miekinia-Błonie, Poland;
- Sulphur–a vulcanizing substance produced by Siarkopol (Tarnobrzeg, Poland).

### 2.2. Preparation of Rubber Nanocomposites

Three compositions were prepared: a mixture of virgin rubber and rubber nanocomposites containing 1 wt% Cloisite Na^+^ or 2 wt% Cloisite 20A. These nanocomposites were selected because of their superior tensile strength, as reported in our previous work [37]. Nanoclay dispersions were prepared with a plasticizer (Santicizer 261A) and then mixed mechanically for 10 min at room temperature, followed by ultrasonic homogenization with a Hielscher UP200H model with an amplitude of 270 µm for 15 min [37]. The plasticization of the compositions was conducted using an industrial two-roller mill WT 300 at 50–60 °C for 10 min. The remaining ingredients were then added to the prepared dispersions and mixed together. The vulcanization was carried out according to PN-ISO 3417:1994 [38] for 15 min using a press PH-2PW90ie at temperatures between 150 and 160 °C and a pressure of 10 MPa. The vulcanizates were left to cool in the molds, and then samples of defined shapes and sizes were cut out for further testing.

### 2.3. Aging

Thermal aging of selected rubber vulcanizates was carried out in a circulating laboratory oven at a temperature of 70 °C for 7, 14, 21, and 28 days in accordance with PN-ISO 188:2000 [39]. Method A. All aged samples were stored for 24 h at 23 °C in order to obtain thermal equilibrium. Additionally, the selected samples were subjected to thermal aging only after the cyclical tensile tests (0–100%, 0–200%, and 0–300%) and then aged.

### 2.4. Evaluation of Mechanical Properties Before and After Aging

The tensile strength and strain at break of the rubber samples were assessed according to the PN-ISO 37:2007 [40] standard at a deformation rate of 200 mm/min using an Instron 5566 machine (Instron Co., Buckinghamshire, UK) equipped with Merlin software v. 22108. Measurements were made both on unaged samples, after thermal aging, and on samples subjected to cyclic stretching and only then aged.

The aging factor (*K_a_*) of the samples was calculated using the following formula:

(1) $$K_{a}=\frac{TS_{ag}\cdot SB_{ag}}{TS_{ba}\cdot {SB}_{ba}}$$

where: *TS_ba_*—Tensile strength before aging, (MPa), *TS_ag_*—Tensile strength after aging, (MPa), *SB_ba_*—Strain at break before aging, (%), *SB_ag_*—Strain at break after aging, (%).

The shape retention coefficient (*K_s_*) was determined in accordance with the PN-ISO 815:1998 [41] standard on a Zwick/Roell Z010 testing machine. The test was carried out at room temperature on aged and unaged cylindrical samples with a diameter of 32 mm. The samples were first compressed by 20% for 3 min, removed, and their height was measured 5 min after removal. The *K_s_* coefficient was calculated using the following formula:

(2) $$K_{s}=\frac{h_{2}-h_{1}}{h_{0}-h_{1}}$$

where: *h*_0_—sample height before the test (mm), *h*_1_—height of the sample after 20% compression (mm), and *h*_2_—height 3 min after removal of the load (mm).

Mullins effect-the measurement involved cyclic stretching, returning the sample at a constant speed of 50 mm/min and increasing the maximum elongation of the sample during the measurement by 100% of the sample length, up to 300%. The study was conducted for compositions 1 and 2 and the reference sample. Samples were tested before and after 7 days of aging on an Instron 5566 testing machine.

### 2.5. Characterization

Fourier-transform infrared spectroscopy was used to check the presence of characteristic groups using a spectrometer Nicolet 6700 (Thermo Fisher Scientific, Waltham, MA, USA), mode ATR with diamond crystal, 64 scans, resolution 4 cm^−1^. 

Thermogravimetric analysis used a analyzer Q500 (TA Instruments) with a temperature profile of 25–1000 °C and a heating rate of 10 °C/min in a nitrogen atmosphere. 

Differential scanning calorimetry: Samples were analyzed using a Stare System (Mettler Toledo) at a scanning rate of 10 °C/min in nitrogen in the temperature range of −70 °C to +130 °C.

X-ray diffraction tests were performed using a Rigaku Miniflex-600 powder diffractometer (Japan) equipped with a Co Kα source radiation (λ = 1.7903 Å) operating at 40 kV, an emission current of 15 mA, and a scanning speed of 8°/min step of 0.2°.

Scanning electron microscope NovaNano SEM 450e (FEI Company, Eindhoven, The Netherlands) was used to analyze the morphology of the samples previously broken in nitrogen, and then covered by a sputter coater with a thin layer of Au/Pd for 60 s.

## 3. Results and Discussion

**Figure 1:**
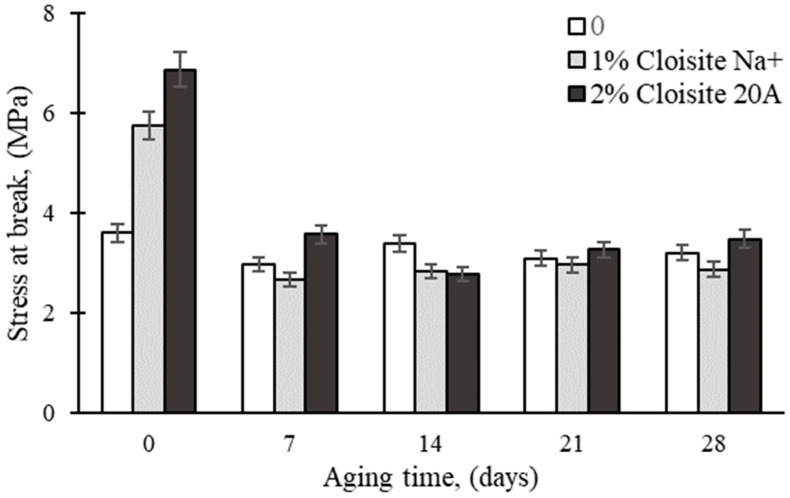
Effect of thermal aging time on the tensile strength of epoxy nanocomposites.

Figure 1 shows the effect of thermal aging on the tensile strength (TS) of rubber nanocomposites prepared from Cloisite 20A and Cloisite Na^+^. It can be seen that the TS decreased significantly after 7 days of aging, regardless of the tested nanocomposites. The lowest TS decrease was shown by the unmodified epoxy matrix (17%), while 50% was assigned to Cloisite 20A and Cloisite Na^+^ nanoclays, respectively. The decrease in TS can be attributed to the breakage of the chemical bonds between the rubber matrix and nanoparticles. Similar results have been obtained by other researchers [11,13,37].

**Figure 2:**
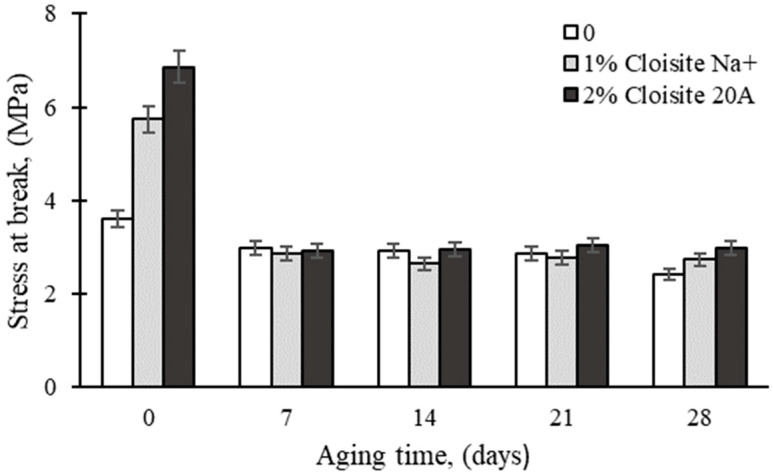
Effect of UV aging time on the tensile strength of epoxy nanocomposites.

As shown in Figure 2, a similar decrease in TS was noted with UV aging of the rubber nanocomposites. As seen above, the lowest decrease was shown by the unmodified epoxy matrix (17%) compared to 57% for the Cloisite 20A-based nanocomposite. Ultraviolet exposure can cause complex chemical changes in rubber nanocomposite samples. As reported by Tan et al. [42], oxidation reactions occur on the surface EPDM rubber. The degree of oxidation increased with increasing aging time; moreover, the results of Wang et al. [43] confirmed that the mechanical properties of the EPDM coating were significantly affected by temperature and aging time.

However, Mishra et al. [44] attributed the decrease in the mechanical and thermal properties of montmorillonite-modified silicone rubber to a decrease in the crosslinking density and an increase in mobility of rubber chains due to prolonged exposure to UV.

**Figure 3:**
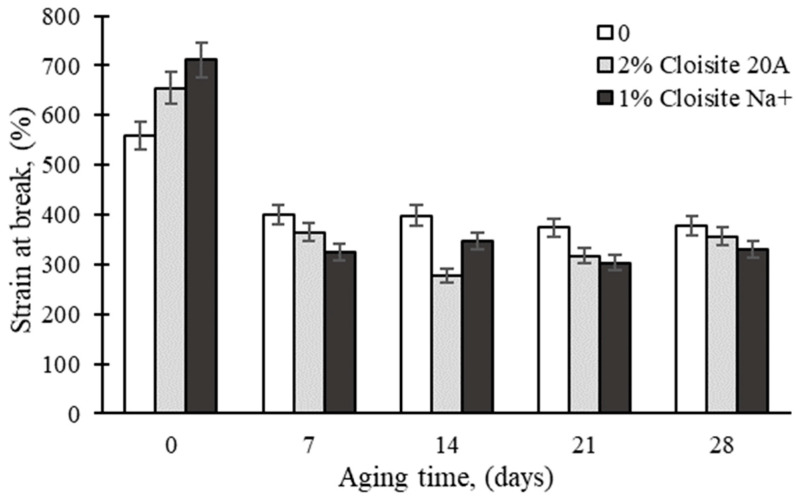
Effect of thermal aging time on tensile strain at break of rubber nanocomposites.

The effect of the thermal aging time on the tensile strain at the break of the rubber nanocomposites is presented in Figure 3. The strain at break of the virgin matrix decreased by approximately 30%, while the decrease in Cloisite 20A and Cloisite Na^+^ reached 46% and 49%, respectively, after one week of aging. Thermal aging leads to the breakage of polymer chains and, consequently, a reduction in their elongation.

**Figure 4:**
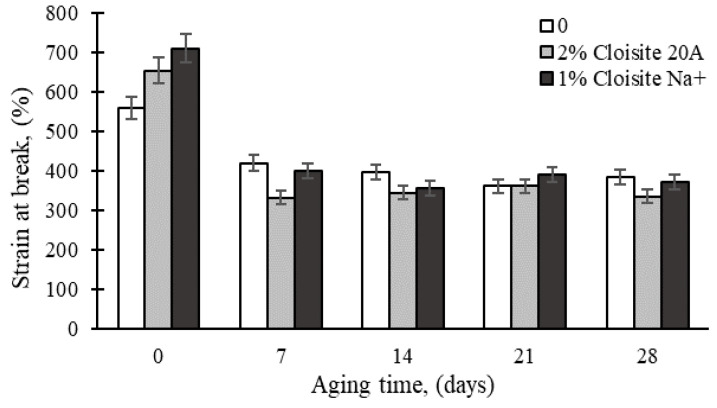
Effect of UV aging time on tensile strain at break of epoxy nanocomposites.

Figure 4 shows the effect of UV aging on the strain at the break of rubber resin modified with Cloisite 20A and Cloisite Na^+^. As in the case of the thermal aging results, a maximum strain decrease of approximately 50% was observed for the Cloisite 20A-based nanocomposite. However, one cannot observe drastic changes after longer aging times. As previously mentioned, most of the sample degradation took place during the first week of aging.

**Figure 5:**
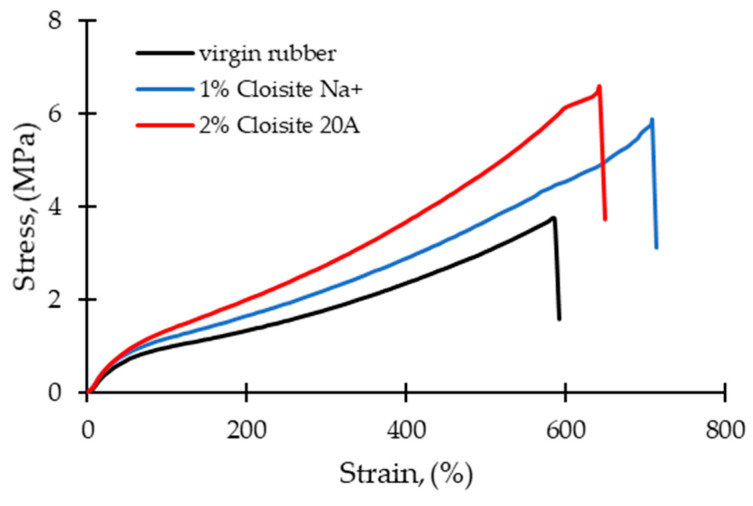
Stress-strain curves of a virgin rubber matrix and rubber nanocomposites containing 1 wt% Cloisite Na^+^ or 2 wt% Cloisite 20A.

Figure 5 shows the stress-strain curves of a virgin rubber matrix and nanocomposites containing 1 wt% Cloisite Na^+^ or 2 wt% Cloisite 20A. The curves were obtained from the raw data of a selected sample of each composition. It was found that the stress and strain at break of the nanocomposites increased with the addition of nanoclay. These results are in agreement with those shown in Figure 1, Figure 2, Figure 3 and Figure 4.

Table 1 shows the mechanical properties of the epoxy nanocomposites before and after 7 days of thermal or UV aging. The samples containing 1 wt% Cloisite Na^+^ or 2 wt% Cloisite 20A were selected for their maximum impact strength. The energy to break evaluated from the stress and the strain increased by about 30% and 40% compared to the virgin rubber matrix for the nanocomposites containing 1 wt% Cloisite Na^+^ and 2 wt% Cloisite 20A, respectively. However, a drastic reduction in the energy to break was noted for both types of nanoclays subjected to either thermal or UV aging. Indeed, the energy to break decreased by more than 95% for the unmodified rubber vulcanizate and rubber nanocomposites. As expected, the values of the moduli at 100% elongation were lower than those at 300% elongation, while both moduli were higher after thermal aging than after UV aging. The values of the modulus at 300% elongation increased by 20%, 15%, and 7% after thermal aging for the unmodified sample, nanocomposites with Cloisite Na^+^, and Cloisite 20A, respectively. As mentioned earlier, the formation of crosslinking after thermal aging necessarily leads to an increase in the stiffness of the sample, whereas UV aging does not affect the bulk of the sample, and thus does not produce more crosslinking and, thus, higher rigidity.

It is seen that thermal aging and UV radiation affect the tensile strength, strain at break, energy to break, and modulus at 100% and 300% elongation of the nanocomposites and virgin vulcanizate differently. This depends on the aging time and temperature, as well as the intensity of UV radiation. The tensile properties of the nanocomposite containing Cloisite Na^+^ were better after UV irradiation than those of the nanocomposite containing Cloisite 20A. The opposite result was obtained for thermal aging due to the barrier effect of the organomodified nanoclay. It is well known that the properties and thermal stability of polymer nanocomposites depend on the type of nanoclays, their surface modification and degree of dispersion, and the level of their intercalation within the matrix. The effect of UV radiation can cause chemical reactions on the surface of the sample, which can lead to deterioration of its properties. However, heat dissipates inside the sample, breaking the various bonds and consequently leading to the degradation of the latter.

The values of the shape retention coefficient (*K_s_*) and aging coefficient (*K_a_*) are presented in Table 2. It is seen that *K_s_* did not change after aging, except for that of the virgin matrix, which decreased by 15% after thermal aging. This confirms the barrier effect of montmorillonite nanoplatelets against heat diffusion, leading to an increase in the thermal stability of the nanocomposites.

Surprisingly, the aging coefficient *K_a_* of the unmodified vulcanizate and nanocomposite based on Cloisite Na^+^ was lower with thermal aging than with UV radiation, while the opposite was obtained with the nanocomposite based on Cloisite 20A.

**Figure 6:**
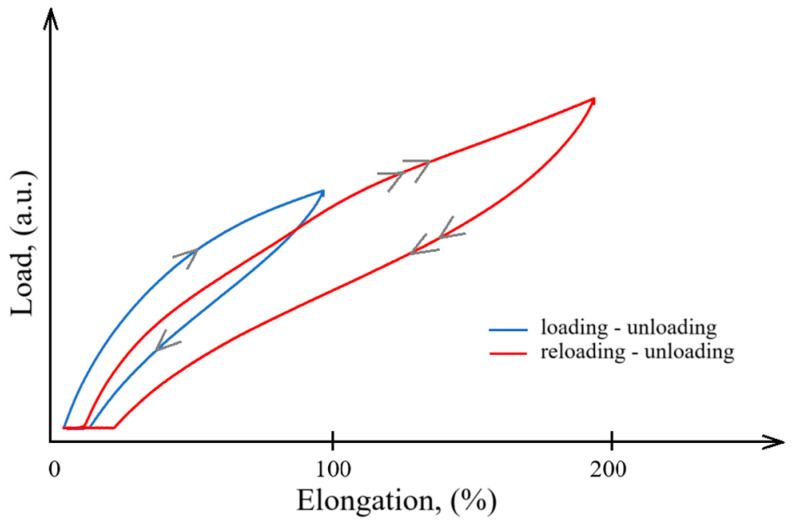
Schematic presentation of the loading-unloading-reloading process.

The loading-unloading process is shown schematically for two cycles in Figure 6, in which the 1st cycle is in blue and the 2nd cycle is in red. Stress softening is accompanied by residual deformation, and the reloading of the rubber sample requires a load lower than the previous loading, thus confirming the Mullins effect [30,32]. This phenomenon is in contrast to the idealized Mullins response with reloading of the sample following the same path as the previous loading [45]. This characteristic behavior has been reported in other works on filled and unfilled rubber materials [46,47,48,49,50].

The energies were calculated between the loading and next reloading curves (designed as loading-reloading energy) as well as from the hysteresis loop (hysteresis energy), and their values are presented for the samples after aging and stretching in Table 3 and Table 4, respectively. Thermal aging was chosen for the evaluation of the energies dissipated during the cyclic stretching of the samples and compared to the aged samples due to the better properties of the samples compared to UV aging.

From Table 4, it can be observed that the energy increased with increasing levels of stretching of the samples before or after aging. Moreover, it was shown that the dissipated energy of the samples that were first cyclically stretched and then aged was slightly greater than that of the samples that were first subjected to thermal ageing, most likely due to the breakdown of the bonds within the matrix and/or the bonds between the latter and the nanoparticles during stretching. Similar results were obtained for other rubber nanocomposite systems [46,47]. Other investigations attributed the stress softening of filled and unfilled rubber vulcanizates to the destruction of the filler aggregates [48,49], chain disentanglements, which resulted in a decrease in the effective crosslink density [50,51,52], chain breakage at the interface between the rubber and the fillers [53,54], and the rupture of chain-filler and chain-chain bonds [55].

Similar to the previous results (Table 3), the values of the loading-reloading energies are shown in Table 4, which increased before or after aging with increasing elongation of the samples. Moreover, it appeared that the level of stress softening was lower when Mullins was applied after aging of the samples. These results are consistent with ours for unaged rubber nanocomposites [44]. Kittur et al. [56] showed that thermo-oxidative aging caused the evolution of crosslinks and entanglements that significantly affect the inelastic response of the elastomers. The results revealed that thermo-oxidative aging induced additional crosslinking accompanied by chain scission, with a predominance of crosslinking over chain scission. Moreover, it was demonstrated that the aging process significantly influenced the stress relaxation of the samples. However, Bouaziz et al. [57] confirmed that the stiffness of carbon black-filled butadiene rubber increased with thermal aging time due to the reorganization of the crosslinks and/or reformation of the network. In addition, they showed that the Mullins was not affected by aging. In another study, it was found that Mullins softening increased with aging time as a result of the increase in the crosslink density of thermo-oxidatively aged elastomers [29,58]. In addition to the type of filler used, its content, and dispersion, it has been shown that the Mullins effect also depends significantly on the adhesion between the filler and matrix [59]. In contrast, Merckel et al. [60] demonstrated that the Mullins softening effect remains unaffected by a change in the crosslink density, but increases with increasing filler content. 

A heat treatment applied to the rubber nanocomposite components that are subjected to deformations would result in reversibility of the Mullins effect and hence ensure their extended service life.

**Figure 7:**
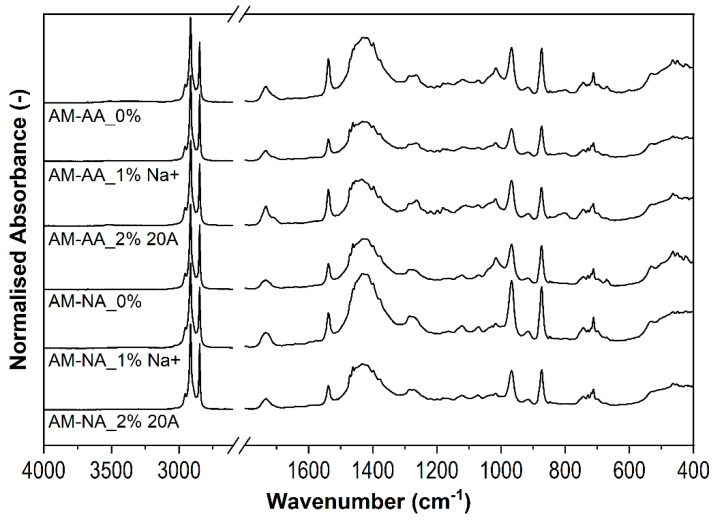
FTIR spectra of samples after Mullins (AM), after aging (AA), and before aging (NA).

Figure 7 shows the FTIR spectra of the rubber samples that were cyclically stretched to 300% according to the Mullins procedure before and after thermal aging. Two peaks appearing at 3000 cm^−1^ and 2850 cm^−1,^ with the same intensity in all spectra, were attributed to the stretching vibrations of the C-H groups in the methylene groups of saturated aliphatic hydrocarbons. A band related to the stretching vibrations of unsaturated C=C groups was identified for all tested samples at 1730 cm^−1^, with the highest band height exhibited by the control and nanocomposite based on 2 wt% Cloisite 20A. The intensity of the band associated with C=C vibrations increased in the unmodified sample after aging, indicating continuous degradation of the elastomer. After the addition of Cloisite 20A, the number of unsaturated groups did not increase in the nanocomposite after the aging process, which suggests a thermal protection effect of Cloisite 20A. This effect is even more visible in the sample containing Cloisite Na^+^, particularly in the unaged sample. The lower intensity of this band compared to sample “0”, confirmed the interaction of Cloisite Na^+^ with unsaturated C=C groups.

The band appeared at 1430 cm^−1^ and was attributed to CH_2_ of the nanocomposites compared to the virgin sample designated “0”, thus proving the barrier effect of the nanoparticles. However, the aged nanocomposites contained fewer CH_2_ groups than the non−aged samples. The decrease in band height correlates with the increase in the content of the previously mentioned band for C=C. Bands from the =CH strain vibrations of unsaturated bonds, whose intensity was significantly less in the nanocomposites than in the matrix itself, were observed in the range of 970–870 cm^−1^ and 720 cm^−1^, independent of the aging and stretching of the sample. This once again proves the thermal protection effect of the modifiers used.

**Figure 8:**
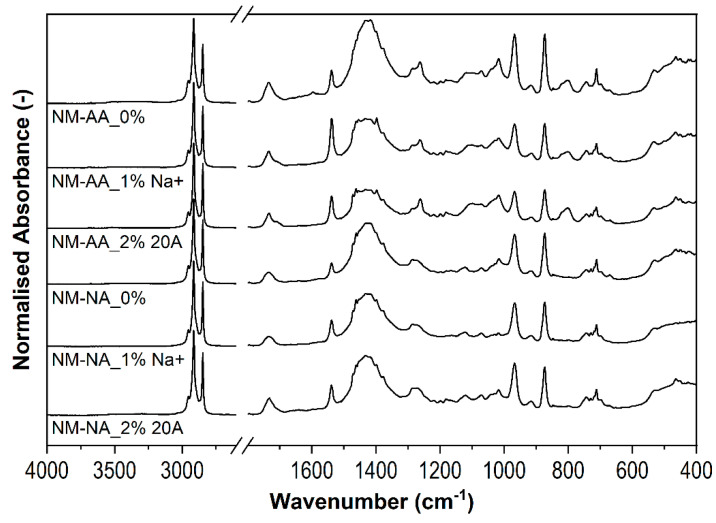
FTIR spectra of samples with no Mullins stretching.

Figure 8 shows the spectra of the unstretched samples before and after thermal aging. In all tested spectra before and after aging, it was noted that the stretching vibrations of the CH group in the CH_2_ groups showed almost identical bands at 3000 cm^−1^ and 2850 cm^−1^, indicating the heat resistance of the rubber matrix. Distinct differences in band intensities were observed at wave numbers 1730 cm^−1^, 1540 cm^−1^ and 1430 cm^−1^. The first band corresponds to the stretching vibrations of the unsaturated C=C bonds with the greatest height for sample “0”, attributed to the greatest degree of aging of the sample. The addition of nanoparticles reduced the number of unsaturated C=C groups in the rubber, which, analogous to the results in Figure 5, may be related to the thermal protection effect of the fillers. Moreover, it is seen that the band of the sample with Cloisite Na^+^ was smaller compared to that of Cloisite 20A, and its reduction can even be seen in the stretched and aged sample. It appears that there may be a better matrix interaction with Cloisite Na^+^ than with Cloisite 20A, and secondary crosslinking of the rubber may occur due to the temperature. The band at 1520 cm^−1^ corresponding to C≡N stretching vibrations was clearly the most intense in the unmodified rubber formulation and decreased in the nanocomposites, which may be related to the shielding effect of nanoparticles with C≡N groups. The large broadband at 1430 cm^−1^ is assigned to the CH_2_ deformation vibrations of the methylene groups. The strongest band intensity was recorded for the sample without nanoclays, which may be explained by the barrier effect of the latter. Among the nanocomposites, the highest concentration of methylene groups was observed in the nanocomposite containing 1 wt% Cloisite Na^+^, which was cyclically stretched. For the remaining stretched samples, the intensity of the band at 1430 cm^−1^ was similar in the unaged samples and those without the nanofiller, and in the unaged sample containing and without Cloisite 20A. This confirms the resistance to heat and stretching of the rubber chains of the samples containing Cloisite Na^+^. Similarly, deformation vibrations of unsaturated =CH bonds were also visible in the ranges of 970–870 cm^−1^ and 720 cm^−1^.

**Figure 9:**
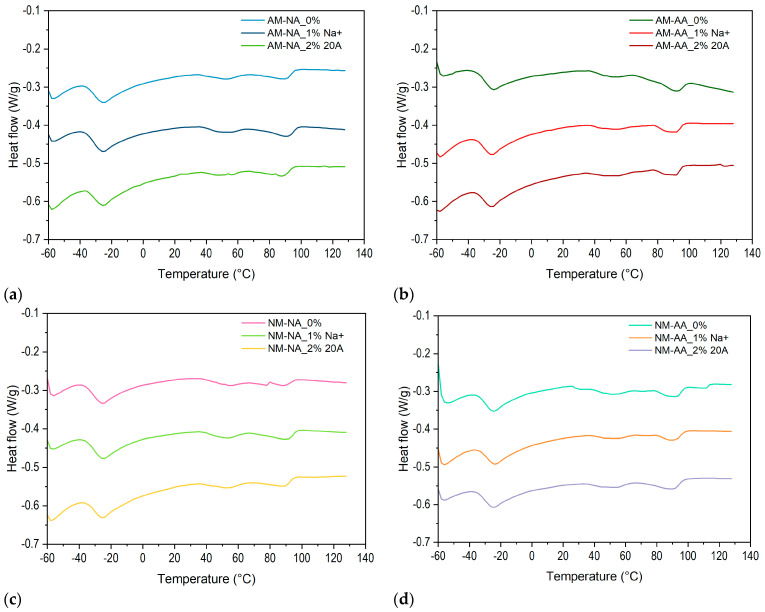
DSC thermograms of rubber samples: (**a**) no aging, after Mullins (NA, AM), (**b**) after aging, after Mullins (AA, AM), (**c**) no aging, no Mullins (NA, NM), (**d**) after aging, no Mullins (AA, NM).

The Differential Scanning Calorimetry thermograms of rubber samples that were cyclically stretched to 300% according to the Mullins procedure before and after thermal aging are shown in Figure 9. Three endothermic peaks of similar intensity were identified in the thermograms for all tested samples, and the associated data are presented in Table 5. The first peak is associated with the glass transition temperature (T_g_), while the peak denoted as T_p1_ is assigned to the crosslinking of the elastomer. The small intensity indicates that the rubber was partially cross-linked. However, peak T_p2_, with a smaller intensity, could be related to the degradation of the sample. As can be seen, the recorded transition temperatures were influenced by many factors, such as the type of nanoparticles, the aging process, and the process of cyclic stretching of the rubber samples.

It can be noted that the incorporated nanoparticles decreased the T_g_ of stretched samples (AM) in the range from −29 to −32 °C and increased it in unstretched samples (NM). However, the added nanoclays and cyclic stretching did not affect the T_g_ of the unaged samples. A slight decrease in T_g1_ was observed in the aged and stretched samples, which may accompany the structure rearrangement that occurs during these operations. Moreover, it was shown that nanoparticles lowered the crosslinking temperature of the rubber matrix (T_p1_) in the unaged samples and increased it in the aged samples. This may be linked to the barrier effect of the nanofillers against the diffusion of heat and, consequently, the degradation of the material. Regardless of the stretching, unaged rubber samples containing Cloisite Na^+^ had a higher crosslinking temperature than those with Cloisite 20A but lower than that of the pure rubber matrix.

We noticed an increase in the temperature T_p1_ in the stretched and aged samples as a result of the addition of nanofillers. This result did not concern unstretched samples, which may be explained by the arrangement of elastomer chains due to the cyclically acting tensile load. As expected, the incorporation of nanoparticles led to an increase in temperature T_p2_, thereby increasing the thermal resistance of the stretched, aged, and unaged composites. This was not observed in the unstretched samples with a more convoluted structure of polymer chains.

**Figure 10:**
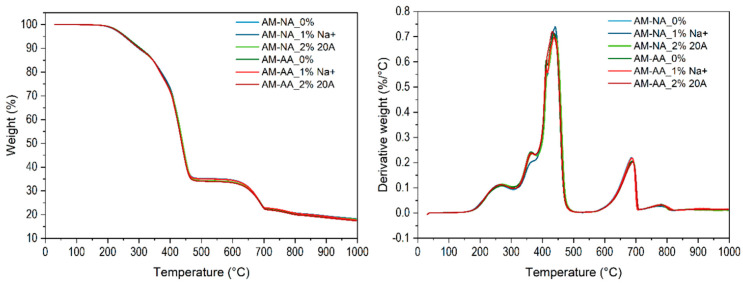
The thermograms showing the change in weight change of aged (AA), unaged (NA), and Mullins stretched samples (AM) as a function of temperature.

Thermogravimetric analysis was used to evaluate the effects of nanoclay content, thermal aging, and cyclic stretching on the thermal stability and degradation temperature of the rubber nanocomposites. Figure 10 shows the weight loss of the unaged, aged, and stretched rubber samples as a function of temperature. Results from TG and DTG curves of the rubber samples are presented in Table 6**.** Unexpectedly, all the aged and unaged samples exhibited similar thermogravimetric and differential thermogravimetric (DTG) curves. This can be explained by the two competitive effects of the nanoclays: a catalytic effect leading to an increase in degradation, and a barrier effect increasing the thermal stability of the nanocomposites. Similar results have been reported elsewhere [61,62]. The decomposition of the rubber matrix can be achieved with alkylammonium cations, which act as surface modifiers of the nanoclay [63,64].

**Figure 11:**
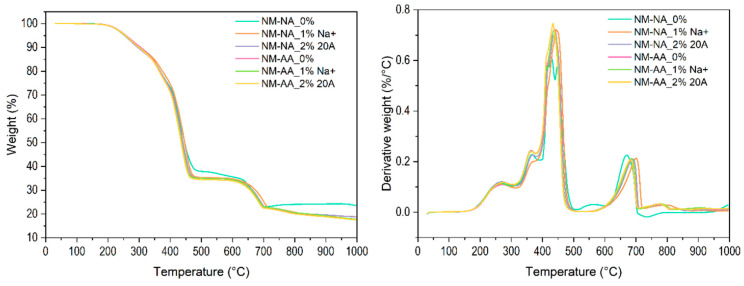
Weight of aged (AA), unaged (NA), and unstretched samples (NM) as function of temperature.

Figure 11 shows the thermogravimetric thermograms of the aged (AA), unaged (NA), and unstretching samples (NM). As mentioned above, for cyclically stretched samples, unstretched (NM) samples subjected to aging (AA) or unaged (NA) exhibited similar thermograms. This can be explained by the reversibility of Mullins stress softening [64,65,66]. It has been reported that cyclically stretched filled or unfilled rubber samples recover their initial properties after heating [64,65] or with time [63,65] as a result of chain relaxation. It should be emphasized that the recovery of the properties of filled or unfilled rubber materials would not lead to a reduction in their service life.

We distinguish three different regions with 9.38–13.62%, 37.36–53.52%, and about 12% of weight loss. The first region between 262 and 367 °C, with a small weight loss, can be related to the decomposition of low-molecular-weight compounds and the release of volatile products. The degradation started in all samples at ~250 °C and ended at ~470 °C. The fastest rate of degradation was noted at about 435 °C, corresponding to maximum weight loss, indicating the very good thermal stability of all tested samples (i.e., stretched, unstretched, aged, and unaged).

**Figure 12:**
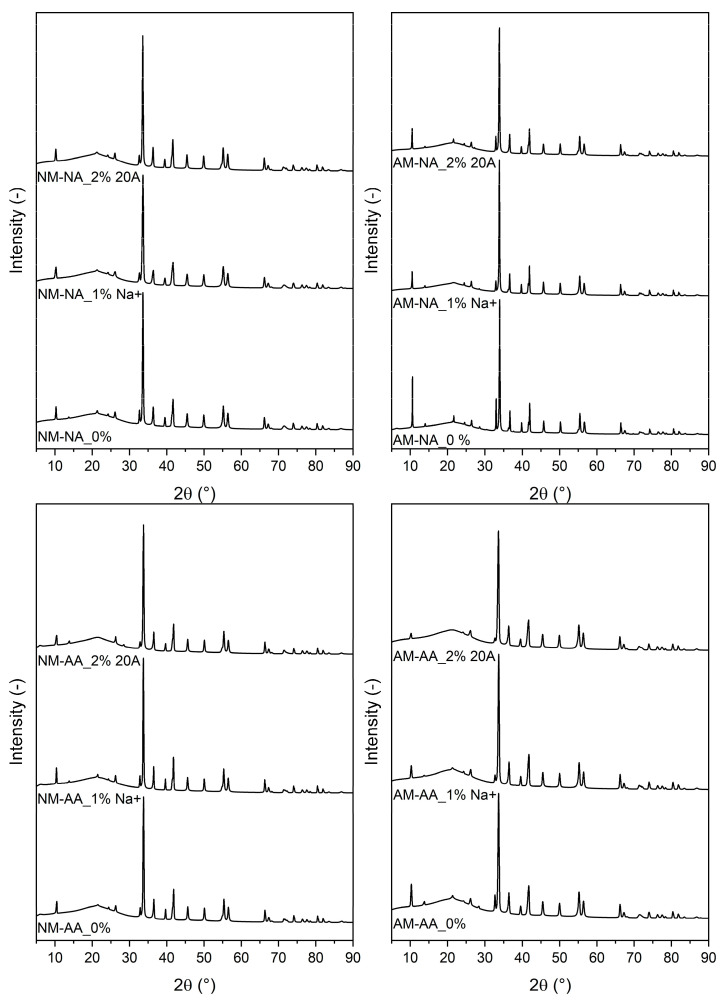
X-ray diffraction patterns of aged and unaged rubber samples, with and without Mullins stretching (NM-NA: no Mullins, no aging; AM-NA: after Mullins, no aging; NM-AA: no Mullins, after aging; AM-AA: after Mullins, after aging).

The X-ray diffraction (XRD) technique was used to analyze the internal structure of a material as well as the intercalated or exfoliated nanoplatelets. Figure 12 shows the XRD patterns of the unaged (NA) rubber samples with Mullins stretching (AM) and without stretching (NM). Aged samples (AA) are also presented with and without cycling Mullins stretching. It is observed that the XRD pattern of the rubber exhibited peaks with varying intensities at 10.2°, 33.6°, 36.3°, and 41.6°. The difference between unmodified hydrophilic nanoclay (Cloisite Na^+^) and organomodified MMT (Cloisite 20A) is also demonstrated. In addition, the peaks of the stretched rubber samples were smaller, regardless of the rubber composition, probably due to the decrease in the interlayer spacing caused by the alignment of the nanoplatelets [34]. This effect is characterized by a decrease in the intensity of the XRD line at 10.2°, indicating changes in the structure of the sample. Surprisingly, the peak at 10.2° of the virgin sample (designated AM-NA 0%) was the most intense and the smallest in the sample with 2% Cloisite 20A (AM-AA and NM-AA). The Mullins effect is associated with the internal structure of rubber becoming increasingly stable after cyclic stretching, leading to consistent material properties [67].

**Figure 13:**
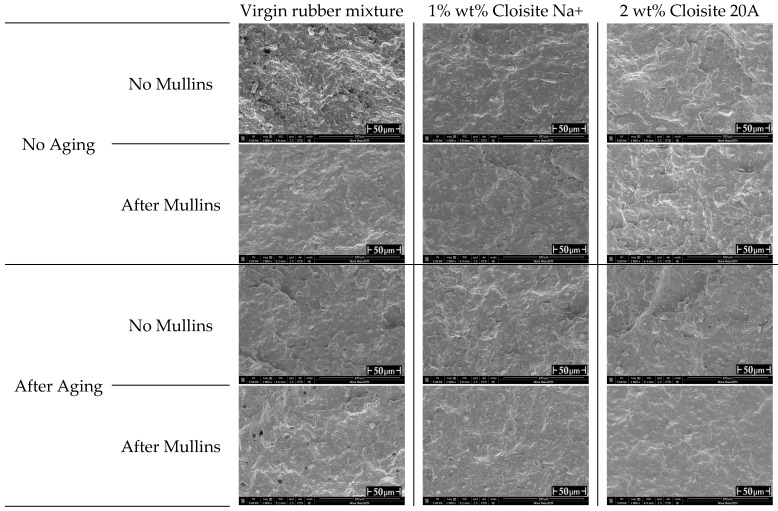
Micrographs of rubber samples before and after aging and before and after cyclic loading.

Figure 13 shows SEM micrographs of the virgin rubber formulation and rubber composites containing 1 wt% Cloisite Na^+^ and 2 wt% Cloisite 20A before and after thermal aging, as well as before stretching and after stretching (Mullins effect). The surface of the virgin unaged sample is rough with some holes, while it is flatter and less rough after aging. It was noticed that the addition of a nanofiller had a positive effect on the barrier properties of samples exposed to elevated temperatures. The phenomenon of separation of the nanofiller from the matrix is smaller, which is illustrated by the lack of microcracks at the matrix-nanofiller interface. Micropores resulting from the aeration of the rubber mixture during its vulcanization are visible in all analyzed samples. The nanoparticles were well embedded in the matrix, with a few oval-shaped pores visible in all tested samples.

In the case of the cyclically stretched samples, microcracks appeared at the matrix–nanofiller interface, and the smaller their number, the higher the concentration of the nanofiller. Microcracks were visible in the stretched samples. After adding nanoparticles, microcracks are fewer and more visible in samples with lower nanofiller content. Heating led to the separation of the nanoparticles from the matrix. However, some structures were ordered in the stretched samples. Moreover, it should be pointed out that microcracks were larger when using a nanofiller with an unmodified surface (i.e., Cloisite Na^+^) than when using an organomodified nanofiller such as Cloisite 20A. This can be related to the occurrence or lack of interaction between the matrix and the nanofiller.

## 4. Conclusions

The following conclusions can be drawn from the results obtained by the combined aging and stress softening of acrylonitrile-butadiene rubber/natural rubber compositions:

- The tensile strength and strain at break decreased by approximately 50% after 7 days of aging, regardless of the tested nanocomposites, due to the breakage of the chemical bonds between rubber and nanoparticles. The shape retention coefficient was not affected by heat for all tested samples except the virgin rubber sample, which exhibited a decrease of about 15% under thermal aging. The aging coefficient Ka of the unmodified vulcanizate and the Cloisite Na^+^ based nanocomposite was smaller with thermal aging than with UV radiation, while the Cloisite 20A nanocomposite exhibited the opposite. The values of the hysteresis and loading-reloading energies increased with increasing levels of sample stretching before or after aging. The dissipated energy of the samples that were first stretched and then aged was slightly higher than that of the samples that were first subjected to thermal aging, probably due to the breakdown of matrix bonds between the latter and the nanoparticles during stretching. Furthermore, the loading-reloading energy results indicated that the level of stress softening was lower when Mullins was applied after the aging of the samples.
- FTIR spectroscopy analysis confirmed the presence of the characteristic groups as well as a decrease in the peak of unsaturated C=C and C≡N groups following the addition of nanoparticles, which can be attributed to the thermoprotective effect of the latter. DSC results indicated a slight decrease in T_g_ was observed in aged and stretched samples and an increase in the temperature of the first endothermic peak due to the addition of nanofillers in the stretched and aged samples. Thermogravimetric analysis revealed that all tested samples exhibited similar thermograms, regardless of their state of stretching or aging. SEM analysis of the virgin unaged sample showed that it was rough with some holes, while it was flatter and less rough after aging. The nanoparticles were well embedded and dispersed in the rubber matrix.
- It has been shown that the Mullins softening effect combined with aging and other parameters affects the safe use of rubber nanocomposite components. An adequate heat treatment applied to the components could result in the reversibility of the Mullins effect and hence ensure their extended service life.

## Figures and Tables

**Table 1:** Mechanical properties of rubber composites after 7 days of thermal and UV aging.

		Tensile Strength, (MPa)	Strain at Break, (%)	Energy to Break, (J)	Modulus at 100% Elongation (MPa)	Modulus at 300% Elongation (MPa)
0	Before aging	3.6	560	158.0	0.99	1.83
	thermal aging	3.0	400	4.7	1.15	2.23
	UV aging	3.0	420	4.5	1.16	2.15
1 wt% Cloisite Na^+^	Before aging	5.7	711	205.5	1.14	2.17
	thermal aging	2.7	325	3.6	1.27	2.52
	UV aging	2.9	400	4.4	1.18	2.21
2 wt% Cloisite 20A	Before aging	6.9	655	222.9	1.33	2.71
	thermal aging	3.6	364	4.9	1.40	2.91
	UV aging	2.9	332	3.9	1.35	2.64

**Table 2:** Values of shape retention coefficient (*K_s_*) and aging coefficient (*K_a_*) before and after 7 days of thermal and UV aging.

Content of Nanoclay	Aging Type	*K_s_*	*K_a_*
		(−)	(−)
0	No aging	0.99	−
	Thermal aging	0.83	0.59
	UV aging	0.96	0.62
1 wt% Cloisite Na^+^	No aging	0.98	−
	Thermal aging	0.98	0.33
	UV aging	0.93	0.43
2 wt% Cloisite 20A	No aging	0.96	−
	Thermal aging	0.99	0.61
	UV aging	0.92	0.46

**Table 3:** Energy (J) between loading and unloading at different levels of elongation.

	Samples Composition								
	Virgin Rubber Sample			1 wt% Cloisite Na^+^			2 wt% Cloisite 20A		
Elongation, (%)	100	200	300	100	200	300	100	200	300
Mullins without aging	0.09	0.28	0.43	0.12	0.30	0.46	0.11	0.31	0.51
Mullins, followed by aging	0.11	0.30	0.43	0.10	0.30	0.45	0.12	0.34	0.53
Aging followed by Mullins	0.11	0.25	0.39	0.09	0.23	0.38	0.11	0.29	0.50

**Table 4:** Energy (J) between the loading and next reloading samples at different stretching cycles.

	Samples Composition								
	Virgin Rubber Sample			1 wt% Cloisite Na^+^			2 wt% Cloisite 20A		
Elongation, (%)	100	200	300	100	200	300	100	200	300
Mullins without aging	0.07	0.17	0.22	0.06	0.17	0.25	0.08	0.19	0.28
Mullins, followed by aging	0.07	0.21	0.25	0.07	0.21	0.27	0.07	0.23	0.33
Aging followed by Mullins	0.06	0.13	0.21	0.06	0.14	0.20	0.07	0.17	0.28

**Table 5:** Results from DSC curves.

Sample	m_0_ [mg]	T_g_ [°C]	T_p1_ [°C]	T_p2_ [°C]
AM-NA_0%	9.79	−31	51	90
AM-NA_1% Na^+^	9.62	−32	49	91
AM-NA_2% 20A	10.09	−31	48	88
AM-AA_0%	9.42	−29	51	91
AM-AA_1% Na^+^	9.86	−30	52	92
AM-AA_2% 20A	9.74	−30	54	91
NM-NA_0%	9.14	−32	55	89
NM-NA_1% Na^+^	9.30	−32	53	91
NM-NA_2% 20A	9.31	−30	54	89
NM-AA_0%	9.91	−31	52	93
NM-AA_1% Na^+^	10.01	−29	55	83
NM-AA_2% 20A	9.72	−30	53	89

m_0_ [mg]…initial weight of the sample, T_g_ [°C]…T_g_ midpoint extracted from 1. Heating: T_p1_ [°C]…Temperature of the first endothermic peak extracted from 1. heating–very small.

**Table 6:** Results of TG and DTG of tested rubber samples.

Sample	m_0_ [mg]	T_1_ [°C]	Δm_1_ [%]	T_2_ [°C]	Δm_2_ [%]	T_3_ [°C]	Δm_3 _ [%]	T_4_ [°C]	Δm_4_ [%]	m_T_ [%]
AM-NA_0%	12.430	262	10.50	366	12.13	436	42.25	686	12.85	81.91
AM-NA_1% Na^+^	12.347	265	11.39	365	shoulder	Sh+441	53.50	687	12.41	81.74
AM-NA_2% 20A	12.880	270	10.66	367	12.54	435	42.57	689	11.87	81.95
AM-AA_0%	12.723	271	9.81	363	12.86	438	42.42	688	12.28	82.50
AM-AA_1% Na^+^	11.872	268	10.55	364	11.51	438	43.01	687	12.08	82.32
AM-AA_2% 20A	13.131	268	10.25	367	12.31	432	43.54	690	11.83	82.62
NM-NA_0%	11.999	260	11.17	366	13.62	430	37.36	Sh+672	14.46	76.45
NM-NA_1% Na^+^	13.605	264	11.27	368	shoulder	Sh+444	53.52	701	12.66	81.14
NM-NA_2% 20A	12.858	266	11.91	367	11.39	437	41.74	689	12.32	81.24
NM-AA_0%	13.318	272	9.38	363	13.63	438	42.02	686	12.25	82.17
NM-AA_1% Na^+^	12.228	266	10.18	363	12.87	437	42.01	687	12.07	82.20
NM-AA_2% 20A	12.440	270	10.45	365	12.88	434	42.27	682	12.00	82.45

m_0_ [mg]…initial weight of the sample, Δm_1_ [%]…the weight at temperature T_1_, peak 1, Δm_2_ [%]…the weight at temperature T_2_, peak 2 (small, in AM-NA 1% Na, NM-NA_1% Na^+^ only shoulder); Δm_3_ [%]…the weight at temperature T_3_, peak 3 (highest, degradation of polymer); Δm_4_ [%]…the weight at temperature T_4_, peak 4, Δm_T_ [%]…the total weight lo.

## Data Availability

Data (including stress-strain data) can be obtained from the authors upon request.

## References

[1] Bokobza L. (2018). Natural rubber nanocomposites: A review. Nanomaterials.

[2] Sethulekshmi A.S., Saritha A., Joseph K. (2022). A comprehensive review on the recent advancements in natural rubber nanocomposites. Int. J. Biol. Macromol..

[3] Sengupta R., Chakraborty S., Bandyopadhyay S.A., Dasgupta S., Mukhopadhyay R., Auddy K., Deuri A.S. (2007). A short review on rubber/clay nanocomposites with emphasis on mechanical properties. Polym. Eng. Sci..

[4] Danafar F., Kalantari M. (2018). A review of natural rubber nanocomposites based on carbon nanotubes. J. Rubber Res..

[5] Srivastava S.K., Mishra Y.K. (2018). Nanocarbon reinforced rubber nanocomposites: Detailed insights about mechanical, dynamical mechanical properties, payne, and mullin effects. Nanomaterials.

[6] Bhattacharya M., Maiti M., Bhowmick A.K. (2008). Influence of different nanofillers and their dispersion methods on the properties of natural rubber nanocomposites. Rubber Chem. Technol..

[7] Archibong F.N., Orakwe L.C., Ogah O.A., Mbam S.O., Ajah S.A., Okechukwu M.E., Igberi C.O., Okafor K.J., Chima M.O., Ikelle I.I. (2023). Emerging progress in montmorillonite rubber/polymer nanocomposites: A review. J. Mater. Sci..

[8] Sayfo P., Pirityi D.Z., Pölöskei K. (2023). Characterization of graphene-rubber nanocomposites: A review. Mater. Today Chem..

[9] Salehiyan R., Sinha Ray S., Sinha Ray S. (2018). Rubber nanocomposites: Processing, Structure–Property Relationships, Applications, Challenges, and Future Trends. Processing of Polymer-Based Nanocomposites: Processing-Structure-Property-Performance Relationships.

[10] Hejazi I., Sharif F., Garmabi H. (2011). Effect of material and processing parameters on mechanical properties of polypropylene/ethylene–propylene–diene–monomer/clay nanocomposites. Mater. Des..

[11] Bakar M., Przybyłek M., Białkowska A., Żurowski W., Hanulikova B., Stoček R. (2021). Effect of mixing conditions and montmorillonite content on the mechanical properties of a chloroprene rubber. Mech. Compos. Mater..

[12] Choudhury A., Bhowmick A.K., Soddemann M. (2010). Effect of organo-modified clay on accelerated aging resistance of hydrogenated nitrile rubber nanocomposites and their life time prediction. Polym. Degrad. Stab..

[13] Bellas R., Diez J., Rico M., Barral L., Ramirez C., Montero B. (2014). Accelerated ageing of styrene–butadiene rubber nanocomposites stabilized by phenolic antioxidant. Polym. Compos..

[14] Chakraborty S., Kar S., Dasgupta S., Mukhopadhyay R., Chauhan N.P., Ameta S.C., Bandyopadhyay S. (2010). Effect of ozone, thermo, and thermo-oxidative aging on the physical property of styrene butadiene rubber-Organoclay nanocomposites. J. Elastom. Plast..

[15] Chen S., Yu H., Ren W., Zhang Y. (2009). Thermal degradation behavior of hydrogenated nitrile-butadiene rubber (HNBR)/clay nanocomposite and HNBR/clay/carbon nanotubes nanocomposites. Thermochim. Acta.

[16] Bakar M., Przybyłek M., Białkowska A., Żurowski W., Hanulikova B., Masař M. (2021). Effect of Aging Conditions and Rubber Waste Content on the Mechanical Properties and Structure of Montmorillonite/Acrylonitrile Butadiene Rubber Nanocomposites. J. Macromol. Sci. B.

[17] Marković G., Marinović-Cincović M.S., Jovanović V., Samaržija-Jovanović S., Budinski-Simendić J. (2012). Gamma irradiation aging of NBR/CSM rubber nanocomposites. Compos. B Eng..

[18] Pubellier P., Robin C. (2023). Molecular-Level Understanding of the Network Structural Changes of Thermo-oxidatively Aged Natural Rubber Nanocomposites. Macromolecules.

[19] Mathew S., Varghese S., Joseph R. (2013). Degradation behaviour of natural rubber layered silicate nanocomposites. Prog. Rubber Plast. Recycl. Technol..

[20] Keloth Paduvilan J., Velayudhan P., Amanulla A., Joseph Maria H., Saiter-Fourcin A., Thomas S. (2021). Assessment of graphene oxide and nanoclay based hybrid filler in chlorobutyl-natural rubber blend for advanced gas barrier applications. Nanomaterials.

[21] Hu H., Jia Z., Wang X. (2022). Aging mechanism of silicone rubber under thermal–tensile coupling effect. IEEE Trans. Dielectr. Electr. Insul..

[22] Kong Y., Chen X., Li Z., Li G., Huang Y. (2023). Evolution of crosslinking structure in vulcanized natural rubber during thermal aging in the presence of a constant compressive stress. Polym. Degrad. Stab..

[23] Zheng W., Zhao X., Li Q., Chan T.W., Zhang L., Wu S. (2017). Compressive stress relaxation modeling of butadiene rubber under thermo-oxidative aging. J. Appl. Polym. Sci..

[24] Li C., Ding Y., Yang Z., Yuan Z., Ye L. (2020). Compressive stress-thermo oxidative ageing behaviour and mechanism of EPDM rubber gaskets for sealing resilience assessment. Polym. Test..

[25] Peng Q., Zhu Z., Jiang C., Jiang H. (2019). Effect of stress relaxation on accelerated physical aging of hydrogenated nitrile butadiene rubber using time-temperature-strain superposition principle. Adv. Ind. Eng. Polym. Res..

[26] Lou W., Xie C., Guan X. (2022). Coupled effects of temperature and compressive strain on aging of silicone rubber foam. Polym. Degrad. Stab..

[27] Ahagon A., Kirino Y. (2006). Aging of black filled rubber under deformation. Rubber Chem. Technol..

[28] Quang N.T., Hung D.V., Chuong B., Le T.T. (2021). Mullins Effect and Crack Growth in Natural Rubber Vulcanizates during Heat Aging and Cyclic Loading. Eng. Technol. Sustain. Dev..

[29] Kittur M.I., Andriyana A., Ang B.C., Ch’ng S.Y., Verron E. (2022). Inelastic response of thermo-oxidatively aged carbon black filled polychloroprene rubber. Part II: Mullins effect. Polym. Degrad. Stab..

[30] Diani J., Brieu M., Vacherand J.M. (2006). A damage directional constitutive model for Mullins effect with permanent set and induced anisotropy. Eur. J. Mech. A Solids.

[31] Dargazany R., Itskov M. (2009). A network evolution model for the anisotropic Mullins effect in carbon black filled rubbers. Int. J. Solids Struct..

[32] Zhu P., Zhong Z. (2021). Constitutive modelling for the mullins effect with permanent set and induced anisotropy in particle-filled rubbers. Appl. Math. Model..

[33] Anssari-Benam A., Akbari R., Dargazany R. (2023). Extending the theory of pseudo-elasticity to capture the permanent set and the induced anisotropy in the Mullins effect. Int. J. Non-Linear Mech..

[34] Chu H., Lin J., Lei D., Qian J., Xiao R. (2020). A network evolution model for recovery of the Mullins effect in filled rubbers. Int. J. Appl. Mech..

[35] Merckel Y., Diani J., Brieu M., Caillard J. (2013). Constitutive modeling of the anisotropic behavior of Mullins softened filled rubbers. Mech. Mater..

[36] Fazekas B., Goda T.J. (2021). Constitutive modelling of rubbers: Mullins effect, residual strain, time-temperature dependence. Int. J. Mech. Sci..

[37] Białkowska A., Przybyłek M., Sola-Wdowska M., Masař M., Bakar M. (2023). Mechanical properties and Mullins effect in rubber reinforced by montmorillonite. Bull. Pol. Acad. Sci. Tech. Sci..

[38] (1994). Rubber—Measurement of Vulcanization Characteristics with the Oscillating Disc Curemeter.

[39] (2000). Rubber, Vulcanized or Thermoplastic—Accelerated Ageing and Heat-Resistance Tests.

[40] (2007). Rubber, Vulcanized or Thermoplastic—Determination of Tensile Stress-Strain Properties.

[41] (1998). Rubber, Vulcanized or Thermoplastic—Determination of Compression Set at Ambient, Elevated or Low Temperatures.

[42] Tan J.H., Chen C.L., Wu J.Y., He R., Liu Y.W. (2021). The effect of UV radiation ageing on the structure, mechanical and gas permeability performances of ethylene–propylene–diene rubber. J. Polym. Res..

[43] Wang S., Xu J., Li H., Liu J., Zhou C. (2022). The effect of thermal aging on the mechanical properties of ethylene propylene diene monomer charge coating. Mech. Time-Depend. Mat..

[44] Mishra S., Shimpi N.G., Mali A.D. (2013). Effect of surface modified montmorillonite on photo-oxidative degradation of silicone rubber composites. Macromol. Res..

[45] Ogden R.W., Roxburgh D.G. (1999). A pseudo–elastic model for the Mullins effect in filled rubber. Proc. R. Soc. London. Ser. A Math. Phys. Eng. Sci..

[46] Pan Y., Zhong Z. (2017). Modeling the Mullins effect of rubber-like materials. Int. J. Damage Mech..

[47] Sasikumar K., Manoj N.R., Mukundan T., Khastgir D. (2016). Hysteretic damping in XNBR–MWNT nanocomposites at low and high compressive strains. Compos. Part B-Eng..

[48] Diani J., Fayolle B., Gilormini P.A. (2009). review on the Mullins effect. Eur. Polym. J..

[49] Krpovic S., Dam-Johansen K., Skov A.L. (2021). Importance of Mullins effect in commercial silicone elastomer formulations for soft robotics. J. Appl. Polym. Sci..

[50] Ma C., Ji T., Robertson C.G., Rajeshbabu R., Zhu J., Dong Y. (2017). Molecular insight into the Mullins effect: Irreversible disentanglement of polymer chains revealed by molecular dynamics simulations. Phys. Chem. Chem. Phys..

[51] Fu W., Wang L., Huang J., Liu C., Peng W., Xiao W., Li S. (2019). Mechanical properties and Mullins effect in natural rubber reinforced by grafted carbon black. Adv. Polym. Technol..

[52] Song Y., Yang R., Du M., Shi X., Zheng Q. (2019). Rigid nanoparticles promote the softening of rubber phase in filled vulcanizates. Polymer.

[53] Khajehsaeid K. (2016). Development of a network alteration theory for the Mullins-softening of filled elastomers based on the morphology of filler–chain interactions. Int. J. Solids Struct..

[54] Qian M., Zou B., Chen Z., Huang W., Wang X., Tang B., Liu Q., Zhu Y. (2021). The influence of filler size and crosslinking degree of polymers on Mullins effect in filled NR/BR composites. Polymers.

[55] Marckmann G., Verron E., Gornet L., Chagnon G., Charrier P., Fort P. (2002). A theory of network alteration for the Mullins effect. J. Mech. Phys. Solids.

[56] Kittur M.I., Andriyana A., Ang B.C., Ch’ng S.Y., Verron E. (2022). Inelastic response of thermo-oxidatively aged carbon black filled polychloroprene rubber. Part I: Viscoelasticity. Polym. Degrad. Stab..

[57] Bouaziz R., Ahose K.D., Lejeunes S., Eyheramendy D., Sosson F. (2019). Characterization and modeling of filled rubber submitted to thermal aging. Int. J. Solids Struct..

[58] Li Z., Xu H., Xia X., Song Y., Zheng Q. (2019). Energy dissipation accompanying Mullins effect of nitrile butadiene rubber/carbon black nanocomposites. Polymer.

[59] Bokobza L. (2023). Elastomer nanocomposites: Effect of filler–matrix and filler–filler interactions. Polymers.

[60] Merckel Y., Diani J., Brieu M., Gilormini P., Caillard J. (2011). Characterization of the Mullins effect of carbon-black filled rubbers. Rubber Chem. Technol..

[61] Castaño-Rivera P., Calle-Holguín I., Castaño J., Cabrera-Barjas G., Galvez-Garrido K., Troncoso-Ortega E. (2021). Enhancement of chloroprene/natural/butadiene rubber nanocomposite properties using organoclays and their combination with carbon black as fillers. Polymers.

[62] Carli L.N., Roncato C.R., Zanchet A., Mauler R.S., Giovanela M., Brandalise R.N., Crespo J.S. (2011). Characterization of natural rubber nanocomposites filled with organoclay as a substitute for silica obtained by the conventional two-roll mill method. Appl. Clay Sci..

[63] Li H., Wang L., Song G., Gu Z.H., Li P., Zhang C.H., Gao L. (2010). Study of NBR/PVC/oMMT nanocomposites prepared by mechanical blending. Iran. Polym. J..

[64] Corby M., De Focatiis D.S. (2019). Reversibility of the Mullins effect for extending the life of rubber components. Plast. Rubber Compos..

[65] Denora I., Marano C. (2024). Stretch-induced softening in filled elastomers: A review on Mullins effect related anisotropy and thermally induced recovery. Polym. Test..

[66] Dargazany R., Itskov M. (2013). Constitutive modeling of the Mullins effect and cyclic stress softening in filled elastomers. Phys. Rev. E.

[67] Gao J., Yang X., Huang L., Suo Y. (2018). Experimental study on mechanical properties of aramid fibres reinforced natural rubber/SBR composite for large deformation–quasi-static mechanical properties. Plast. Rubber Compos..

